# Assessment of ownership of smart devices and the acceptability of digital health data sharing

**DOI:** 10.1038/s41746-024-01030-x

**Published:** 2024-02-22

**Authors:** Md Mobashir Hasan Shandhi, Karnika Singh, Natasha Janson, Perisa Ashar, Geetika Singh, Baiying Lu, D. Sunshine Hillygus, Jennifer M. Maddocks, Jessilyn P. Dunn

**Affiliations:** 1 Department of Biomedical Engineering, Duke University, Durham, NC USA; 2 Duke University Health System, Durham, NC USA; 3 Department of Political Science, Trinity College of Arts & Sciences, Duke University, Durham, NC USA; 4 Sanford School of Public Policy, Duke University, Durham, NC USA; 5 Duke University, Department of Biostatistics & Bioinformatics, Durham, NC USA

**Keywords:** Lifestyle modification, Decision making

## Abstract

Smart portable devices- smartphones and smartwatches- are rapidly being adopted by the general population, which has brought forward an opportunity to use the large volumes of physiological, behavioral, and activity data continuously being collected by these devices in naturalistic settings to perform research, monitor health, and track disease. While these data can serve to revolutionize health monitoring in research and clinical care, minimal research has been conducted to understand what motivates people to use these devices and their interest and comfort in sharing the data. In this study, we aimed to characterize the ownership and usage of smart devices among patients from an expansive academic health system in the southeastern US and understand their willingness to share data collected by the smart devices. We conducted an electronic survey of participants from an online patient advisory group around smart device ownership, usage, and data sharing. Out of the 3021 members of the online patient advisory group, 1368 (45%) responded to the survey, with 871 female (64%), 826 and 390 White (60%) and Black (29%) participants, respectively, and a slight majority (52%) age 58 and older. Most of the respondents (98%) owned a smartphone and the majority (59%) owned a wearable. In this population, people who identify as female, Hispanic, and Generation Z (age 18–25), and those completing higher education and having full-time employment, were most likely to own a wearable device compared to their demographic counterparts. 50% of smart device owners were willing to share and 32% would consider sharing their smart device data for research purposes. The type of activity data they are willing to share varies by gender, age, education, and employment. Findings from this study can be used to design both equitable and cost-effective digital health studies, leveraging personally-owned smartphones and wearables in representative populations, ultimately enabling the development of equitable digital health technologies.

## Introduction

Technological advancements in both hardware and software are revolutionizing digital devices, which are enabling the development of smaller, more powerful, more sophisticated, and less costly smart portable digital devices. Smart devices—smartphones and smartwatches—are being rapidly adopted by the general population, with 85%^1^ and 31%^2^ of Americans currently owning smartphones and smartwatches, respectively. This widespread penetration of smart devices in the general population has brought forward an opportunity for individuals, researchers, and clinicians to use the large volumes of physiological and activity data collected by these devices in naturalistic settings to monitor health and track disease. For example, individuals can track a variety of personal metrics, such as steps taken, distance traveled, sleep patterns, and heart rate, to maintain or improve their health by better understanding their habits and behaviors and identifying potential modifications. Similarly, researchers and clinicians can use smartphone apps or wearable devices to track the health and behaviors of study participants and patients in their everyday lives, rather than relying on self-reported data or data collected in a laboratory setting. This “real-world” data (RWD) can provide a more accurate and detailed picture of how people behave and respond to different interventions. RWD collected from smart devices and real-world evidence (RWE) generated through RWD analysis have the potential to revolutionize various aspects of healthcare, including outpatient care, clinical trials, and longitudinal remote healthcare monitoring among others. For instance, wearables are being widely used for clinical trials focusing on remote monitoring of patients with chronic diseases, e.g., cardiovascular diseases^3^, cancer^4,5^, and diabetes^6^.

In particular, a new study design for digital health studies has become increasingly popular in recent years, which is known as bring-your-own-device (BYOD). While traditional study designs focus on providing devices or resources to the study participants during the study period to collect data, BYOD studies leverage data collected from individuals’ personally owned smart devices. The COVID-19 pandemic also led to an increase in digital health research studies using the BYOD model, as researchers sped up efforts to detect illness early, provide medical care remotely, and prevent further spread by leveraging data from individuals’ personally owned devices^7–9^. The use of digital health tools for health care has also seen a boom, with a greater number of healthcare providers interested in learning more about such tools and using them for patient care^10^.

Despite the potential of digital health, existing studies suffer from multiple challenges, including the difficulty of drawing population inferences. Lack of diversity in research involving smart device-based algorithms for health monitoring can lead to tools (devices and algorithms) that work well for one population group but not another^11^. For example, significant concerns were raised regarding the inferior performance of the pulse oximeter in people with darker skin tones compared to lighter skin tones^12–15^, pointing towards a larger problem of medical device evaluation being conducted primarily on white individuals^16^. Furthermore, RWE generated using artificial intelligence (AI), which is frequently employed to analyze large amounts of RWD to generate health insights, can produce biased results if the data used for training the AI model is not representative of the population where the model is intended to be used^17^. This challenge is particularly relevant in BYOD studies, where the recruitment pool is limited to people who already own a device and are willing to share their personal data with the research team. The lack of diversity in such studies can hence lead to incorrect predictions for certain segments of the population, particularly for underserved and underrepresented communities.

For this reason, it is very important to understand the ownership and usage of smart devices in the general population or in specific patient populations where the technology will ultimately be deployed, as well as the willingness of people to share their personal data for research purposes. However, minimal research has been conducted in this space to date^18–25^. To investigate the ownership, usage, and willingness to share data from smart devices among patients, we conducted a survey study among a large and diverse sample of representative group of patients from the Duke University Health System (DUHS), which is the Southeast’s preeminent health care provider, with nearly 67,000 inpatient stays and nearly 4.7 million outpatient visits in 2021^26^. We further explored the role of socioeconomic and other demographic factors in smart device ownership, usage, and willingness to share personal data for research purposes. Finally, we compared our results with previously reported results from similar studies to highlight the common and disparate findings from our study.

## Results

We conducted a survey study among a large and diverse sample of patients from DUHS, called Duke Health Listens (DHL)^27^. The survey consisted of 14 questions (Supplementary Document 1) in total. The survey contained single and multiple choice questions to quantify the ownership and usage of smart devices, frequency of usage, reasons for usage or reason for not owning a wearable, willingness to participate in digital health studies, willingness to share data collected by smart devices for research purposes, and type of data the respondents are comfortable sharing and why. We further queried the highest level of education and employment status. Additional data made available by the DHL group included demographic information about respondents, including gender, age group/generation, race/ethnicity, County, and State (North Carolina/ Virginia). The survey was sent out to all DHL patient advisory group members (*N* = 3021) between January 18–30, 2022; 1368 responded (45% response rate). Table 1 describes the characteristics of the survey respondents. A total of 871 (64%) were female, 826 (60%) identified as white, 390 (29%) identified as Black, 60 (4%) identified as Asian, 78 (6%) identified as Hispanic, and about half of the respondents (52%) were age 58 and above.

**Table 1:** Respondent Demographics and Characteristics

	Total (1368)	Male (478)	Female (871)
Race/Ethnicity			
White	784	326	449
Black	390	83	306
Asian	58	26	31
Hispanic	70	31	39
Prefer not to answer	45	6	32
Others^*^	21	6	14
Age Group			
18–25	24	4	17
26–41	176	52	116
42–57	460	121	334
58-76	579	224	355
77+	129	77	49
Highest-Level of Education			
Graduate degree	515	192	314
College graduate	545	186	353
Some college but no degree	231	70	158
High school graduate	74	28	45
Employment Status			
Employed full-time	630	206	417
Employed part-time	108	29	76
Retired, not looking for work	400	190	207
Disabled, not able to work	120	30	85
Not employed, but looking for work	41	14	27
Not employed, not looking for work	69	9	59

Others^*^: American Indian and/or Alaska Native, Native Hawaiian and/or other Pacific Islander, another race/ethnicity.

### Smart device ownership

**Fig. 1:**
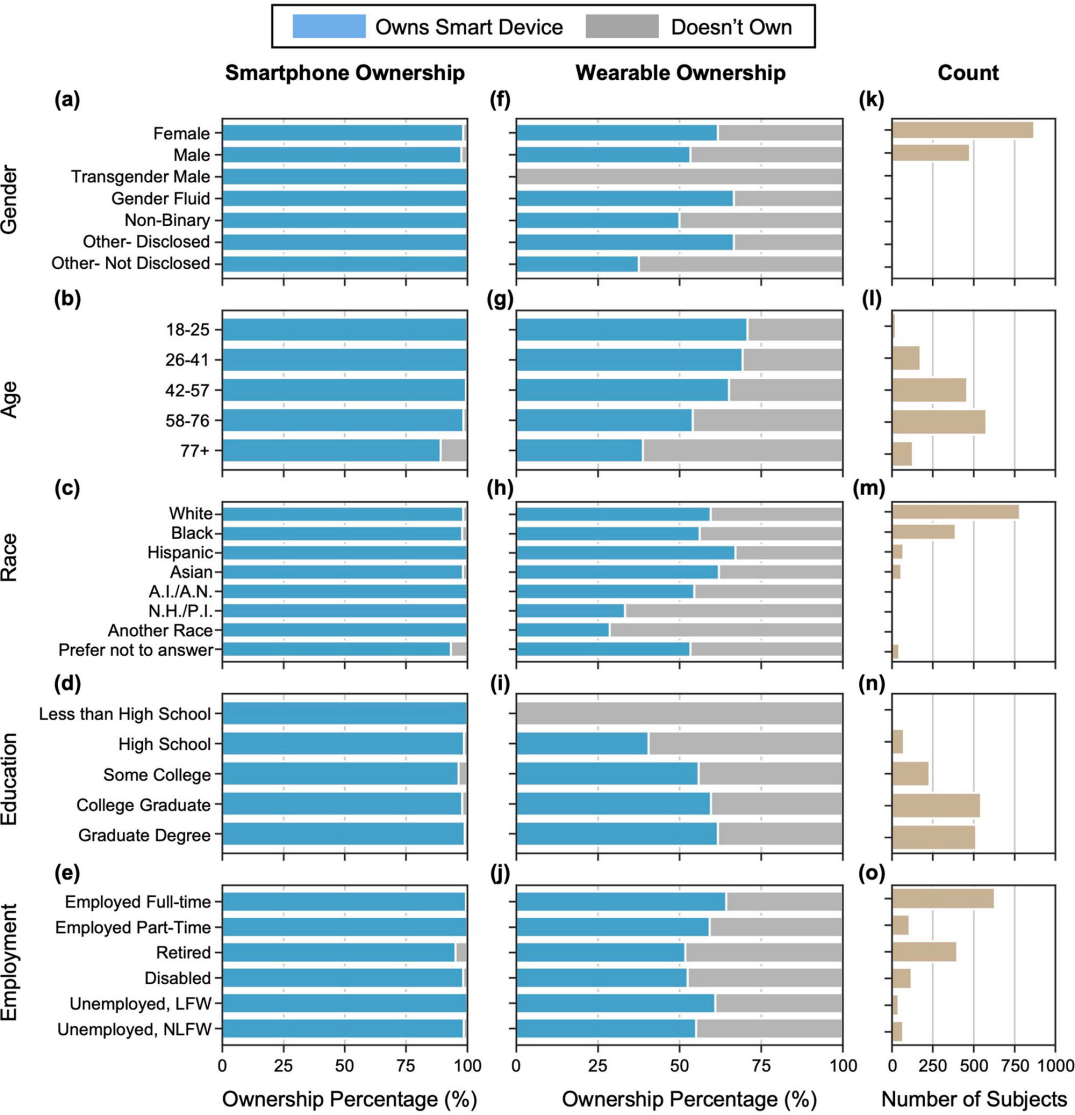
Who owns smart devices?. Smart device ownership: smartphone (**a**–**e**) and wearables (**f**–**j**) ownership by different demographic factors (gender, age, race, highest level of education, and current employment status, respectively), including the number of respondents per demographic group (**k**–**o**). A.I. American Indian, A.N. Alaska Native, N.H. Native Hawaiian, P.I. other Pacific Islander, LFW looking for work, and NLFW not looking for work.

Of the 1368 respondents, 1343 (98%) owned a smartphone, with 894 (66.6%) owning an iPhone and 446 (33.2%) owning an Android device. Smartphone ownership in this population was higher than that reported by both the American Community Survey (2018)^28^ and Pew Research Center (2021)^1^, in which 84% and 85% of respondents reported owning smartphones, respectively. Supplementary Table 1 shows the relative comparison of smartphone and wearable ownership across different demographics between the Pew Research Study^1,29^ and our study. We found that younger people tended to have higher smartphone ownership than older people, and employed people tended to have higher ownership than those who were retired. While we observed statistically significant differences in smartphone ownership across age (*X*^2^(4, *N* = 1368) = 68, *p* < 0.0001) and employment status (*X*^2^(5, *N* = 1368) = 28.3, *p* < 0.0001) (Fig. 1 and Table 2), no substantial differences were observed across gender, race/ethnicity, and education level. Specifically, there was significantly lower ownership in people age 77+ (89%) as compared with younger age groups (age 26–41: 100%; age 42–57: 100%; age 58–76: 98%) (post hoc pairwise comparison with Benjamini Hochberg multiple hypothesis correction *p* < 0.0001). Further, smartphone ownership among retirees (95%), although high in general, was significantly lower than ownership among full-time employees (99.5%) (*p* < 0.001).

**Table 2:** Smart Device Ownership Across Different Demographics

	Total	Owns a Smartphone	Owns a Wearable
Gender			
Male	478	467 (98%)	255 (53%)
Female	871	857 (98%)	538 (62%)
Chi-square (Degrees of Freedom [df])		0.82 (1)	9 (1)
*P* value		0.37	<0.01
Race/Ethnicity			
White	784	771 (98%)	467 (60%)
Black	390	382 (98%)	219 (56%)
Asian	60	57 (98%)	36 (62%)
Hispanic	78	70	47 (67%)
Chi-square (df)		1.5 (3)	3.6 (3)
*P* value		0.68	0.31
Generation (Age Group)			
18–25	24	24 (100%)	17 (71%)
26–41	176	176 (100%)	122 (69%)
42–57	460	458 (100%)	300 (65%)
58–76	579	570 (98%)	313 (54%)
77+	129	115 (89%)	50 (39%)
Chi-square (df)		68 (4)	44 (4)
*P* value		<0.0001	<0.0001
Highest-Level of Education			
Graduate degree	515	510 (99%)	318 (62%)
College graduate	545	534 (98%)	60 (60%)
Some college but no degree	231	223 (97%)	129 (56%)
High school graduate	74	73 (99%)	30 (41%)
Chi-square (df)		5.7 (3)	13 (3)
*P* value		0.12	<0.01
Employment Status			
Employed full-time	630	627 (100%)	405 (64%)
Employed part-time	108	108 (100%)	64 (59%)
Retired, not looking for work	400	381 (95%)	207 (52%)
Disabled, not able to work	120	118 (98%)	63 (53%)
Not employed, but looking for work	41	41 (100%)	25 (61%)
Not employed, not looking for work	69	68 (99%)	38 (55%)
Chi-square (df)		28.3 (5)	18.4 (5)
*P* value		<0.0001	<0.01

In terms of the type of smartphone ownership, there were statistically significant differences between individuals who own an iPhone vs an Android device across race/ethnicity (*X*^2^(3, *N* = 1263) = 11.1, *p* = 0.012), education (*X*^2^(3, *N* = 1320) = 33.5, *p* < 0.0001) and employment status (*X*^2^(5, *N* = 1323) = 37.5, *p* < 0.0001). Specifically, we found significantly higher (*p* < 0.01) iPhone ownership in White vs. Black individuals (70% vs. 60%), those with college or graduate degrees vs. those with high school degrees or some college (69–72% vs. 50–53%), and those with full-time and part-time employment vs. their demographic counterparts. On the contrary, no significant difference was seen in the type of smartphone ownership across gender and age.

Nearly 60% of survey respondents (802 out of 1368) owned a wearable device, which is higher than the numbers reported by Pew Research Center^29^, National Cancer Institute’s Health Information National Trends Survey (HINTS)^24^ and Statista^2^, in which 21%, 30%, and 31% of respondents owned wearable devices, respectively. The most-owned wearable device was the Apple Watch (44.14%, *N* = 354), followed by the Fitbit (42.02%, *N* = 337). Females, younger generations, people with college and graduate degrees, and people with full-time employment had higher ownership compared with their demographic counterparts (Table 2). Specifically, females had a significantly higher (*p* < 0.01) wearable ownership than males (62% vs. 53%), individuals with college (60%) or graduate degrees (62%) had a significantly higher (*p* < 0.01) wearable ownership than individuals with high school degrees (41%), and individuals with full-time employment had a significantly higher (*p* < 0.001) wearable ownership (64%) compared to retired individuals (52%). Wearable ownership was inversely related to age, with those aged 77+ (39%) having significantly lower (*p* < 0.01) wearable ownership as compared with younger age groups (age 18–25: 71%, age 26–41: 69%, age 42–57: 65%, and age 58–76: 54%). Further, those aged 58–76 had significantly lower (*p* < 0.01) wearable ownership than those aged 42–57 (54% vs. 65%). No significant differences were found in wearable ownership by race/ethnicity, which is similar to the results reported by Chandrasekaran et al.^24^ on the 2019 HINTS data. Details of the statistical test results supporting these differences are, for gender, *X*^2^(1, *N* = 1349) = 9, *p* < 0.01); age (*X*^2^(4, *N* = 1368) = 44, *p* < 0.0001); education (*X*^2^(3, *N* = 1365) = 13, *p* < 0.01); employment (*X*^2^(5, *N* = 1368) = 18.4, *p* = 0.0024). Taken together, these findings may help to explain the consistent demographic differences in participation seen in large-scale BYOD digital health studies^9,30,31^.

### Smart device usage

Understanding why and how people use smartphones and wearables can shed light on how easy it may be for researchers and clinicians to obtain physiological and behavioral RWD that would be sufficient for drawing robust research conclusions or effectively implementing health interventions for patients.

**Fig. 2:**
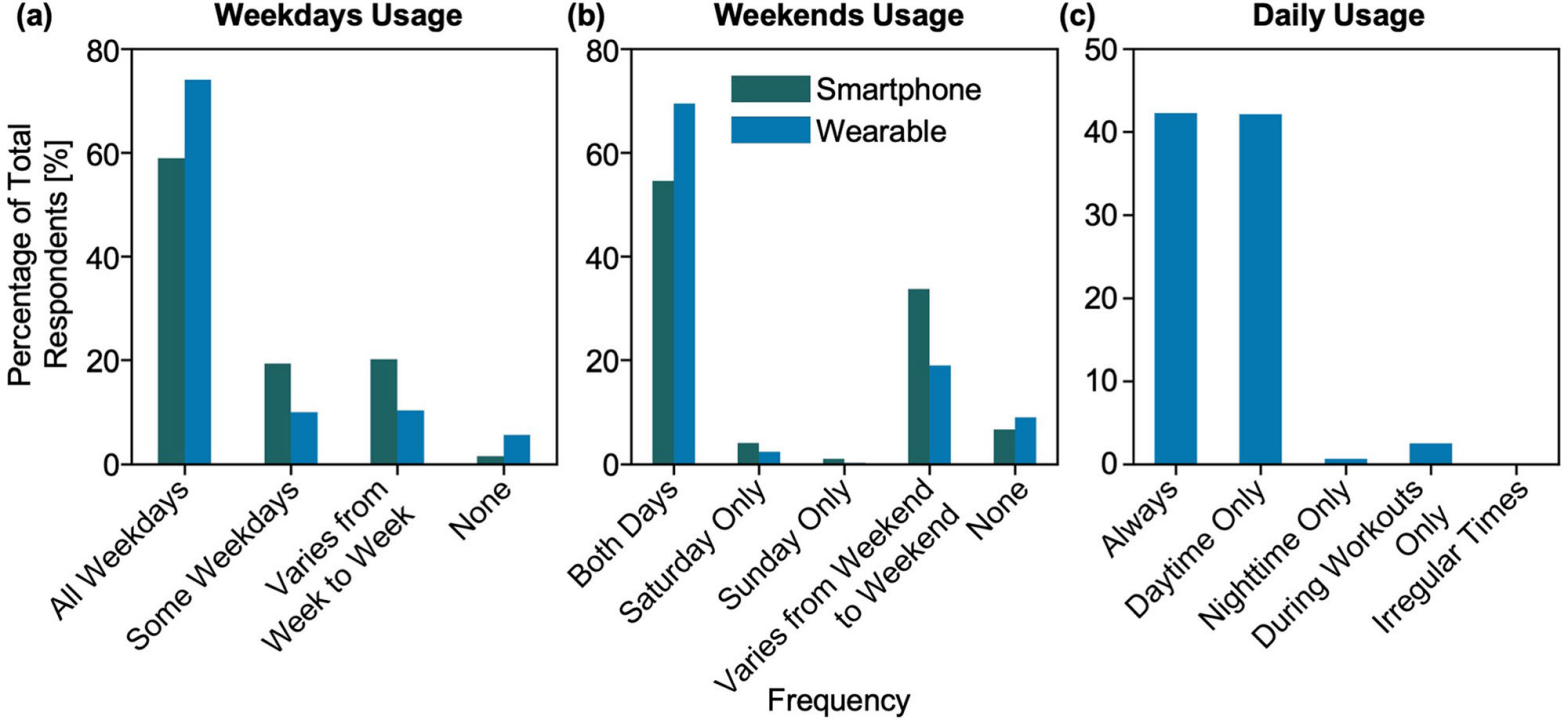
Frequency of usage of smart devices. Usage of smartphones (for activity tracking) and wearables on (**a**) weekdays and (**b**) weekends. (**c**) Daily usage of wearables.

Of the 1343 respondents who own a smartphone, 792 (59%) reported using their smartphones for health and fitness tracking. While there were significant differences (*p* < 0.01) in smartphone usage for health and fitness tracking across age, education, and employment status, no differences were seen across gender and race. A higher percentage of individuals in younger age groups, with college and graduate degrees, and full-time employees reported using their smartphones for health and fitness tracking as compared with their demographic counterparts. Frequencies of smartphone usage for activity tracking during both weekdays and weekends were similar (*p* > 0.05) across gender, age, race, education, and employment, with the majority of people using their smartphone for activity tracking evenly across all weekdays and weekends (Fig. 2a and b).

**Fig. 3:**
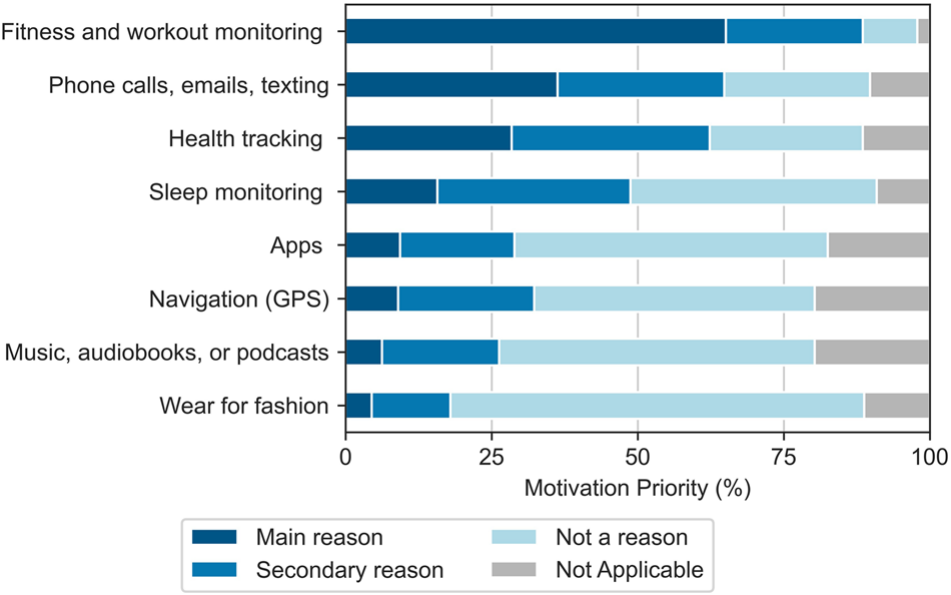
Why do people use wearables?. Motivation for using wearable devices among participants who own them (*N* = 802).

The primary reason for owning a wearable device across all demographic groups was fitness and workout monitoring (heart rate, step tracking, jogging, etc.) (65%, *N* = 522) (Fig. 3). This primary reason for ownership varied significantly (*p* < 0.05) across age, race, and education. The second most common primary reason was sending and receiving phone calls, emails, texts, and messages (36%, *N* = 291). For the majority of respondents, the most common secondary reason for owning a wearable device was health tracking (blood oxygen, heart rhythm, women’s health functions such as ovulation tracking, etc.) (34%, *N* = 272), followed by sleep monitoring (33%, *N* = 265). Only a small number of people reported owning wearables primarily for fashion (4.5%, *N* = 36), and the majority of those who did are Apple Watch owners (69%, *N* = 25). These results highlight that the data of most interest in digital health studies and clinical care, including fitness and workout monitoring, health tracking, and sleep tracking, are likely to be present in BYOD participants as these functionalities are the primary motivation for users owning and using these devices in the first place.

**Fig. 4:**
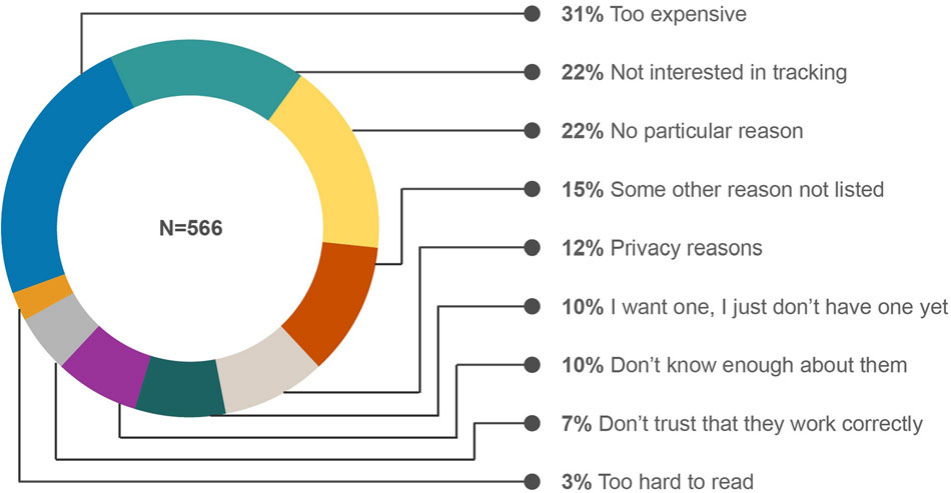
Reason for not owning wearable devices. Reasons for not owning wearable devices among participants who don’t own them (*N* = 566).

In addition to why people *do* own wearable devices, it is also very important to understand why people *don’t* own wearable devices. Such information may highlight factors that can impact the representation of different demographics in a study population, particularly with BYOD study designs, but perhaps even extending beyond to studies that supply devices to participants, for example, if there were discomfort with potential surveillance. Here, we found that the top three reasons for not owning wearable devices were their cost (31%, *N* = 178), lack of interest in tracking (22.4% *N* = 127), and no particular reason (22.08%, *N* = 125) (Fig. 4). The cost of the device was the most important factor for not owning wearable devices across all demographic factors, except gender, where this factor was more important to females as compared with males (37% (*N* = 123) vs 23% (*N* = 52), respectively, *p* < 0.001). This observation points to BYOD designs as potentially exacerbating unequal representation in study populations. The lack of interest in tracking was the second most important factor for not owning wearables across all demographic factors except race and ethnicity, where this reason for not owning a device was more prevalent in White, Asian, and Hispanic as compared to Black individuals (26%, 29%, 24% vs. 11% respectively, *p* < 0.001). This is important to note as intrinsic motivation for tracking may lead to increased adherence to device wear. On the other end, privacy concerns were the fifth most important factor overall (12%, *N* = 68) for not owning wearables, which varied significantly (*p* < 0.05) across age, race, education, and employment. Particularly, privacy concerns were more important for not owning wearables for White than Black respondents (15% vs. 4%, *p* < 0.01), for respondents with graduate and college degrees than respondents with some college experience with no degrees (17% and 14% vs. 2%, respectively, *p* < 0.05), and for respondents with full-time employment than retired respondents (18% vs. 6%, *p* < 0.01). Such factors driving people’s decisions not to own wearable devices can shed light on both why data may be nonrepresentative and what changes could be made in existing research and care ecosystems to address concerns to improve equitability.

Understanding how people use wearables may improve our understanding of the availability and quality of wearable data that can be used for research or healthcare purposes. The context of data collection matters, for example, if people only wear their devices while exercising vs. wear their devices all the time, the average heart rate and activity values would be dramatically different. Furthermore, the circumstances of data collection can affect data availability and accuracy. For example, some devices only collect heart rate variability during sleep. In our study, the majority (>50%) of wearable device owners reported using their device(s) on all weekdays and weekends (Fig. 2a and b), which is similar to the HINTS data (where 47% and 25% of the respondents used wearable every day and “almost every day”, respectively)^24^. However, the frequency of usage over weekdays and weekends varied significantly (*p* < 0.05) across gender, race, and employment. Although a higher percentage of females own wearable devices than males (62% vs. 53%), self-reported wear time was found to be higher in males than females (82% vs. 71% for all weekdays and 78% vs. 66% for all weekends). This is in contradiction to similar research by Pew, where women were more likely than men to say they regularly use their devices (25% vs. 18%)^29^. Also different from the Pew study, we found that the frequency of wearable usage varies across racial and ethnic groups, with a higher percentage of Hispanic people reporting irregular wear times compared to White, Asian, and Black individuals (55% vs. 78%, 78%, and 71%, respectively, for all weekdays and 55% vs. 73%, 69%, and 66% respectively, for all weekends). In contrast, Pew observed higher self-reported regular wear time in Hispanics as compared to Whites and Blacks (26% vs. 20% and 23% respectively)^29^.

Wearables are increasingly being used for studying sleep patterns and behaviors, given that sleep influences overall health. However, device wear during sleep is less prevalent due to factors like comfort and removal for charging, which impacts device use for overall health monitoring. Evaluating device usage during the daytime and nighttime can reveal important insights into how digital health studies can be better designed to monitor sleep and other digital biomarkers collected during sleep. Of wearable device owners, 42.3% reported wearing their devices all the time (day and night), followed by daytime only (42.1%), irregular times (7%), during workout only (3%), and nighttime only (1%) (Fig. 2c). These proportions varied significantly (*p* < 0.05) across gender, race, education, and employment. Upon further investigation across exclusively Fitbit and Apple Watch owners, we found that the majority of Apple Watch owners (63%) use their device during the daytime only, whereas the majority of Fitbit owners (64%) use their device during both the day and nighttime. This observation might relate to the more frequent need for charging for the Apple Watch compared to Fitbit, making Apple Watch users more likely to remove their devices during sleep for charging. Researchers and clinicians may consider such factors when designing and conducting digital health studies involving sleep tracking and other digital biomarkers measured at night and/or during sleep.

### Willingness to participate in digital health studies and to share personal digital data

Willingness to share data is a key factor in understanding which data types are likely to be available for research or clinical purposes. Lack of available information regarding factors related to data sharing can result in biased study data or inequitable clinical practices that serve some, but not all, people. In our study, among the respondents owning smart devices (*N* = 1345), 50%, 32%, and 18% responded “Yes”, “Maybe”, and “No”, respectively, on their willingness to share the personal data collected by their smart devices for research purposes (Table 3). Of the 1102 people who are or may be willing to share their data, 702 (64%) own wearable devices. Willingness to share activity data for future research among wearable owners is slightly higher in our study (57% Yes, 30% Maybe, and 12% No) than previously reported values by Pew Research Center (53% acceptable, 18% not sure, and 29% unacceptable)^29^, Seltzer et al. (where ~40% of respondents were willing to share wearable data immediately, and ~75% agreed to donate wearable data after death)^18^, and Hirst et al. (where roughly one-third of the respondents were willing to share smartphone and wearable data for health research)^21^. Willingness to share wearable data among wearable owners in our study is comparable to previously reported values in the 2019 HINTS data as reported by Rising et al.^22^, where 82% and 70% of respondents were willing to share wearable data with healthcare providers and family or friends, respectively. However, the HINTS study particularly asked about sharing wearable health data with healthcare providers and family or friends, whereas our study focused on willingness to share wearable activity data for research purposes. In our study, participants’ willingness to share their personal data for research purposes varied significantly across age and retirement status– younger generations are more willing and retired individuals are less willing to share their personal data than their demographic counterparts (*X*^2^(8, *N* = 1344) = 28.6, *p* = 0.0004) and employment (*X*^2^(10, *N* = 1345) = 21.5, *p* = 0.018). These findings are similar to those of Pew^29^, which demonstrated a higher acceptance of wearable data sharing with medical researchers in younger generations than older generations (47% vs 35%), although the overall acceptability for all age groups is higher in our study.

**Table 3:** Willingness to participate in future research studies involving sharing activity data from smart devices

	Yes	Maybe	No
Total	672 (50%)	430 (32%)	243 (18%)
Gender			
Male	247 (53%)	132 (28%)	89 (19%)
Female	415 (48%)	291 (34%)	152 (18%)
Chi-square (df)	4.6 (2)		
*P* value	0.102		
Race/Ethnicity			
White	386 (50%)	249 (32%)	137 (18%)
Black	197 (52%)	123 (32%)	62 (16%)
Asian	27 (47%)	16 (28%)	15 (26%)
Hispanic	35 (50%)	20 (29%)	15 (21%)
Chi-square (df)	4 (6)		
*P* value	0.67		
Generation (Age Group)			
18–25	16 (67%)	7 (29%)	1 (4%)
26–41	92 (52%)	53 (30%)	31 (18%)
42–57	259 (57%)	126 (28%)	73 (16%)
58–76	260 (46%)	206 (36%)	104 (18%)
77+	45 (38%)	38 (32%)	34 (29%)
Chi-square (df)	28.5 (8)		
*P* value	< 0.001		
Highest-Level of Education			
Graduate degree	258 (51%)	160 (31%)	92 (18%)
College graduate	260 (49%)	177 (33%)	98 (18%)
Some college but no degree	122 (54%)	69 (31%)	33 (15%)
High school graduate	31 (42%)	22 (30%)	20 (27%)
Chi-square (df)	7.3 (6)		
*P* value	0.29		
Employment Status			
Employed full-time	324 (52%)	188 (30%)	115 (18%)
Employed part-time	53 (49%)	37 (34%)	18 (17%)
Retired, not looking for work	162 (42%)	143 (34%)	78 (17%)
Disabled, not able to work	75 (64%)	30 (25%)	13 (11%)
Not employed, but looking for work	23 (56%)	13 (32%)	5 (12%)
Not employed, not looking for work	35 (51%)	19 (28%)	14 (21%)
Chi-square (df)	21.4 (10)		
*P* value	0.02		

**Fig. 5:**
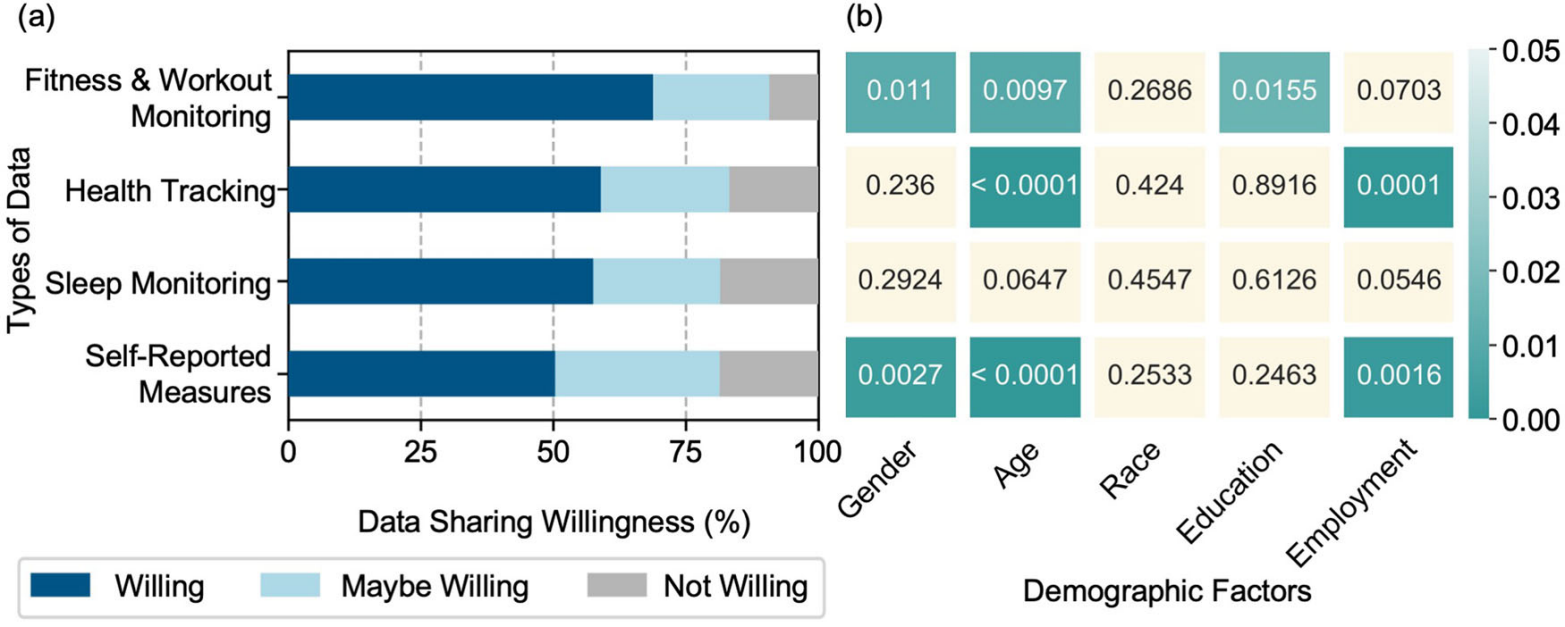
Willingness to share smart device data. **a** Participants willingness to share their smart devices’ data types: Fitness and workout monitoring, health tracking, sleep monitoring, and self-reported measures. **b** Association of participants’ willingness to share different types of data collected by smart devices with demographic factors, with beige color representing *p*-values > 0.05 and green colors representing *p* values < 0.05.

Of participants who are willing to or would consider sharing their personal data for research purposes (*N* = 1102), most are willing to share fitness and workout monitoring data (heart rate, step tracking, jogging, etc.) (69% Yes, 22% Maybe, and 9% No) (Fig. 5). Conversely, the data type that was the least amenable to being shared was self-reported measures from health and fitness apps (e.g., mindfulness and mood, water intake, food logs, women’s health, etc.) with responses of 50% Yes, 31% Maybe, and 19% No. Comfort around sharing different data types for research purposes varied (*p* < 0.05) by gender, age, education, and employment. For example, females were more willing to share fitness and workout monitoring data and self-reported measures from health and fitness apps than their male counterparts (92% vs. 89% and 84% vs. 76%, respectively). Overall, data sharing acceptability decreases with age across all data types. Similarly, retired individuals are less comfortable sharing their personal data compared to full-time and part-time employees. On the contrary, progressing education has demonstrated higher comfort in sharing all data types. There were no differences in the comfort of sharing these data types across races (*p* > 0.05).

## Discussion

Data collected by smart devices are essential for monitoring health and well-being in the real world. However, a lack of knowledge on factors that impact ownership, usage, and willingness to share data from smart devices for research or care, can undermine the ability to draw accurate inferences from research studies. With this study, we sought to close these knowledge gaps. The findings from this study can improve the design of digital health studies and clinical trials to make them more inclusive and equitable.

Factors such as age, education, occupation, economic status, fitness levels, health conditions, etc. are known factors that influence wearable device adoption^32^. Here, we observed a higher proportion of smart device owners in our patient sample compared with nationally representative samples^1,29,33^, which may be attributed to the differences in demographic breakdown among our population vs. nationally representative samples. For example, compared to Pew Research study^29^, our study population had a higher percentage of female participants (56% vs. 64% for Pew Research vs. our study, respectively), a higher proportion of Black participants (11% vs. 29% Black, 73% vs. 57% White, and 17% vs. 5% Hispanic, respectively), and more highly educated (35% vs. 6%with high school or less, 28% vs. 17% with some college experience, and 38% vs. 77% with college and graduate degrees, respectively). Most of the respondents to our survey are more highly educated than the general US population^34^.

In comparison to studies with nationally representative samples^1,24,25,29^, we observed similar trends in smart device ownership, particularly wearables, with a higher percentage of females, younger people, college graduates, employed persons, and people of Hispanic ethnicity owning wearables as compared with their demographic counterparts. However, the overall percentage of the respondents owning wearable devices for each of these demographic groups was higher in our study. Higher wearable ownership in females than males, as observed in our study and in other studies with nationally represented samples, might be a potential factor in the higher participation of females in digital health studies, e.g., AllofUs^30^. Overall, in our study, gender, age, education, and employment status were associated with wearable ownership, and age and employment status were associated with smartphone ownership. Researchers should consider these factors while designing their studies to be representative of the population or of a specific population based on the target end-user of the technology that they are developing or validating.

Greater health awareness among consumers is the main driver of smartwatch adoption^32^, which was also found in our study. Multiple research studies have demonstrated that wearable device owners are significantly more physically active compared to nonowners^25,35–38^. Aside from wearable owners, a majority of smartphone owners (59%) in our study also use their phones for health or fitness tracking. While smartphones can measure some health metrics such as steps and sleep, smartphones and wearables are often used for different primary purposes. Smartphones are much more ubiquitously used for communication purposes, whereas wearables are primarily used for monitoring fitness, activity, and health. The varying purposes are likely to impact ownership and frequency of usage of smartphones and wearables. Ultimately, we found the cost to be the driving force behind not owning a wearable, which shows how socioeconomic disparities can hinder the design of equitable BYOD studies. For that reason, if researchers can develop tools that work well on both smartphones and wearables, this would enable inclusive study designs and technology development. We also found that privacy concerns limiting wearable ownership vary across race, age, education, and employment, where it is more important in White than Black, younger than older, participants with college and graduate degrees than some college experience with no degrees, and full-time employees than retired individuals. Researchers should consider these concerns while designing digital health studies using wearables.

In this study, a majority of respondents reported using their smartphone and wearables for activity monitoring during both weekdays and weekends, which is very important in monitoring individual physiology and behavior over time without needing to account for gaps in the data. However, self-reported wear time of wearables was found to be higher in males than females in our study, which contradicts similar research by Pew^29^. Moreover, the self-reported wear times in our study are significantly higher compared to the 2019 HINTS study^24^ and Pew Research study^29^ (~70-80% vs. 48% and ~20%, respectively), which will require further investigation. To explore the accuracy of such self-reported information, we plan to compare self-reported wear time against smartwatch data collected from the respondents in a subsequent future study. These differences in self-reported wear times might be attributed to the demographic differences between our sample and the 2019 HINTS study and Pew Research study as well. Another key finding of our study is self-reported usage of wearables during daytime and nighttime. Nearly half of respondents (45%) only use their wearables during the day, and the majority of these respondents are Apple Watch owners. Sleep and physiological biomarkers (resting heart rate, respiration rate, SPO2, temperature, etc.) collected during sleep using wearables are important factors in research and clinical care, but such reported wear habits skewed toward daytime data collection will limit digital health technology development progress.

Although findings from our study confirm some of the previous findings from studies with nationally representative population (e.g., Pew Research Study^29^ and HINTS study^24^) on the ownership and usage of wearable devices, these studies with nationally representative population did not ask for certain key details, e.g., types of smart devices the participants own, reasons for not owning wearables, and wearable usage in the daytime vs. nighttime and weekdays vs. weekends. These key details are very important to understand the type and amount of data that can be expected from the study population, and such information should impact digital health study design and the corresponding choice of algorithm. Researchers should consider these important points for designing digital health studies, particularly, BYOD studies.

Another major finding is on willingness to participate in research studies that involve sharing digital health data. Overall, the majority of respondents (82%) would like to or would consider sharing personal activity data with researchers. Interestingly, but perhaps not unexpectedly, willingness to share data varied by the specific data types: willingness to share fitness and workout monitoring data was highest, followed by health tracking, sleep monitoring, and self-reported measures. A key point to note is that these survey responses were collected before the US Supreme Court decided to overturn the constitutional right to abortion (i.e. Roe vs. Wade)^39^. Following this decision, individuals’ perspectives and willingness to share personal data, particularly surrounding women’s health (e.g., ovulation and period tracking), for research or healthcare purposes may now be different^40,41^.

One limitation of this study is the lack of knowledge on the fitness or physical activity levels and comorbidities of respondents. Fitness level is known to impact the use of wearables^25,36,38^, whereas comorbidities might be a driving force for owning and using wearables, for example, people with diabetes who may use a smartwatch to more easily monitor their blood glucose levels reported by a continuous glucose monitor^38^. Since the majority of participants in this are DUHS patients, they may already have health concerns that might affect fitness levels and usage of smart devices, or they might want or need to use smart devices to monitor their health. Thus, the resultant usage of wearables in this population may be biased by specific necessity. We also did not inquire about the reason for the lack of willingness to share wearable data, which is an important point to gain more information on. Another limitation of our study is that we used existing demographic data collected previously by the DHL committee, and thus were limited in the variable ranges to what has already been collected. For example, race/ethnicity were collected in a combined category and we could not collect these variables separately; other demographic classifications are not the most up-to-date with standards or collecting demographic information^42,43^. We also could not collect the income levels of the respondents due to concerns from the DHL committee, which might have provided additional insights for this study.

Future studies should focus on understanding more detail on the comfort levels and concerns around sharing smart device data for research purposes. Such research can support user-centered study design which can increase public trust in digital health research. Avenues for disseminating information regarding such research studies which maximize recruitment reach and make efforts to address public trust are also important for more inclusive study designs. Another key future area to investigate is participants’ willingness to share other data types along with smart device data for research purposes. For example, several digital health studies focus on questionnaire responses on medication use, food logging, symptoms, sleep, etc. These questions, usually conveyed periodically using emails, texts, and/or calls, can serve as ground truth or supplementary information to augment and label data collected from wearables and improve the accuracy of wearable-data-based algorithms. Further studies are needed to evaluate public comfort around such surveys, including the frequency and question types, which could impact willingness to participate in digital health studies and may be addressed through incentivization.

Finally, this manuscript aims to provide information to researchers, clinicians, wearables companies, and other digital health stakeholders on optimizing the design of research studies and clinical trials using smart devices with a representative population that will help validate the reliability, generalizability, and equitable use of these technologies both from a hardware and AI perspective. Proactively designing digital health studies with consideration for demographic and socio-economic factors to include representative populations will improve the utility of smart devices and RWD for monitoring health and disease, and further augment clinical decision-making and remote home monitoring.

## Methods

### Participant recruitment and data collection

We conducted a survey study among a large and diverse sample of patients from the Duke University Health System (DUHS), called Duke Health Listens (DHL). DHL is an online community (with 3021 members at the time of our study, 95% of whom are patients from DUHS), where the members can offer direct feedback via online surveys on ways to improve the patient experience, ideas for new services or offerings, how to enhance DUHS’s online platforms, and feedback on health care messaging and marketing programs^27^. This feedback helps providers and staff of DUHS create the best experience possible in Duke Health hospitals and clinics. Only adults aged 18 and older are allowed to be DHL advisors, and participation is not restricted to past or current DUHS patients.

We launched this survey study on January 18, 2022, and concluded it on January 30, 2022. The survey consisted of 14 questions (Supplementary Document 1) in total. The survey contains single and multiple choice questions to quantify the ownership and usage of smart devices, frequency of usage, reasons for usage or reason for not owning a wearable, willingness to participate in digital health studies, willingness to share data collected by smart devices for research purposes, and type of data the respondents are comfortable sharing and why. We further queried the highest level of education and employment status. Additional data made available by the DHL group included demographic information about respondents, including gender, age group/generation, race/ethnicity, County, and State (North Carolina/ Virginia).

### Ethics oversight

The participants of DHL provided their informed e-consent (using the Alida platform, Duke Health) when they joined this community to participate in studies organized by DHL. No separate consent was acquired for this specific purpose. The study was determined to be exempt from Institutional Review Board review by the Duke Health Institutional Review Board (Protocol ID: Pro00115157). The survey was prepared in collaboration with the DHL leadership. The DHL leadership conducted the survey study and provided the de-identified responses to the study team upon the completion of the survey.

### Data processing and analysis

Following data collection, the responses and demographic information were analyzed through statistical analysis to understand how different demographic factors are associated with ownership and usage of smart devices as well as participants’ willingness to take part in digital health studies and/or share personal data for research purposes.

The demographic covariates employed for data visualization and analysis were as follows: gender, age group, race/ethnicity, highest level of education, and employment status. A detailed list of groups for each of the demographic factors is provided in Supplementary Document 2. As we had limited representation from gender categories aside from ‘Male’ and ‘Female’ (*N* = 19; detailed breakdown of gender identities not disclosed to preserve participant privacy), we only included binary gender in this analysis. For race/ethnicity categories, we had limited representation from ‘American Indian and/or Alaska Native’, ‘Native Hawaiian and/or other Pacific Islander’, and ‘Another race/ethnicity’ groups (*N* = 11, 3, and 7, respectively), hence, we excluded these categories from our statistical analyses exploring race/ethnicity. For the highest level of education categories, we had limited representation from individuals with ‘Less than high school’ education (*N* = 3), hence, we excluded this category from our statistical analyses exploring education.

### Statistical analysis

We performed chi-square tests of independence to investigate potential associations between different demographic factors (gender, age group, race/ethnicity, highest level of education, and employment status) and our outcome variables of interest (e.g., participants’ ownership, usage, and reason for owning/not owning smart devices, and their willingness to share data from these devices). Following the chi-square tests, we performed post hoc testing using Benjamini-Hochberg multiple hypothesis correction for pairwise comparison of the demographic factors, which demonstrated statistically significant associations with the outcome variables. Alpha was set to 0.05 for all statistical analyses, and degrees of freedom, sample size, test statistics, and *p* values are reported for all statistical results. Total number and percentage of individuals are also reported for the descriptive statistics. Python (version 3.8.5) was used to perform all analyses and generate all figures.

### Reporting summary

Further information on research design is available in the Nature Research Reporting Summary linked to this article.

### Supplementary information

Supplementary Files

Reporting Summary

## Data Availability

The de-identified survey dataset generated and/or analyzed for the current study will be submitted 1 year from the publication date to the Big-Ideas-Lab (BIL) GitHub repository (https://github.com/Big-Ideas-Lab/bil-dhl-survey-analysis) under the title BigIdeasLab_DHL_Survey_Study_1.

## Code availability

The code used for this manuscript is available on the Big-Ideas-Lab (BIL) GitHub repository (https://github.com/Big-Ideas-Lab/bil-dhl-survey-analysis).
