# Supplementary material for: Assessment of ownership of smart devices and the acceptability of digital health data sharing
Source: NPJ Digit Med. 2024 Feb 22;7:44. doi: 10.1038/s41746-024-01030-x (PMC10883993; doi:10.1038/s41746-024-01030-x)
Supplement: Supplementary file 1 — Supplementary Files [file 41746_2024_1030_MOESM1_ESM.pdf]

Supplementary Document 1: Duke Health Listens Survey Questionnaire

This survey asks questions about your interest in smart devices that track your physiological and activity data. The information gathered will be used to help design future studies with Duke patients to see how these devices might positively impact overall health. Please note, you will see 1 or 2 questions about education/occupation so that we ensure we are thinking about supporting health in a way that works for everyone. Thank you for taking the time to complete this survey.

1. Do you own a smartphone? (Check all that apply.)
   a. Yes-iPhone (iApple/iOS)
   b. Yes-Android (Samsung Galaxy, Google Pixel, OnePlus, Motorola, Nokia)
   c. Yes, some other smartphone (please provide)
   d. No
2. Do you currently use your smartphone for any health or fitness tracking? (Check all that apply.)
   a. No
   b. Fitness and workout monitoring (heart rate, step tracking, jogging, etc.)
   c. Health tracking (blood oxygen, heart rhythm, women's health such as ovulation tracking, etc.)
   d. Sleep monitoring (sleep duration, sleep quality, etc.)
   e. Other activity tracking (please describe)
3. How many weekdays (Monday through Friday) do you typically use your **smartphone** for health or fitness tracking?
   a. None
   b. Some weekdays
   c. All weekdays
   d. Varies from week to week
4. Which weekend days (Saturday and Sunday) do you typically use your **smartphone** for health or fitness tracking?
   a. None
   b. Saturday only
   c. Sunday only
   d. Both Saturday and Sunday
   e. Varies from weekend to weekend
5. Do you own any of the following wearable devices? (Check all that apply.)
   a. None
   b. Fitbit
   c. Apple Watch
   d. Garmin
   e. Samsung
   f. Polar
   g. Suunto
   h. Withings

    i. *WITHit*
    j. *Oura ring*
    k. *Whoop*
    l. *Other (please provide)*

6. What would you say are reasons why you do not own a wearable device, such as a smartwatch or activity tracker? Check all that apply.
    a. *I want one, I just don't have one yet*
    b. *No particular reason*
    c. *Too expensive*
    d. *Too hard to read*
    e. *Don't trust that they work correctly*
    f. *Don't know enough about them*
    g. *Not interested in tracking*
    h. *Privacy reasons*
    i. *Some other reason not listed (please provide)*
7. *What* would you say your reasons are for owning a wearable device?

	Main reason	Secondary reason	Not a reason	Not applicable to my device(s)
Apps (social media, news, etc.)				
Fitness and workout monitoring (heart rate, step tracking, jogging, etc.)				
Sleep monitoring (sleep duration, sleep quality, etc.)				
Health tracking (blood oxygen, heart rhythm, women's health such as ovulation tracking, etc.)				
Phone calls, emails, texting				
Music, audiobooks, or podcasts				
Navigation (GPS)				
Wear for fashion				

8. How many weekdays (Monday through Friday) do you typically **wear your wearable device(s)**?
    a. *None*
    b. *Some weekdays*
    c. *All weekdays*
    d. *Varies from week to week*
9. Which weekend days (Saturday and Sunday) do you typically **wear your wearable device(s)**?
    a. *None*
    b. *Saturday only*
    c. *Sunday only*
    d. *Both Saturday and Sunday*

    e. *Varies from weekend to weekend*

10. At what time of day do you typically **wear your wearable device**?
    a. *Always (day and night)*
    b. *Daytime only*
    c. *Nighttime only*
    d. *During workouts only*
    e. *Irregular times (no set times)*
11. What is your occupation or profession?
    a. *Architecture, Engineering*
    b. *Armed forces*
    c. *Arts, Design, Entertainment, Sports, Media*
    d. *Building & Grounds Cleaning, Maintenance*
    e. *Business, Financial Operations*
    f. *Community, Social Services*
    g. *Computer, Mathematical Construction, Extraction*
    h. *Education, Training, Library*
    i. *Farming, Fishing, Forestry*
    j. *Food Preparation, Serving*
    k. *Healthcare Practitioner, Technical Occupations*
    l. *Healthcare Support*
    m. *Installation, Maintenance, Repair*
    n. *Legal*
    o. *Life, Physical, Social Science*
    p. *Management Office, Administrative Support*
    q. *Personal Care, Service*
    r. *Production*
    s. *Protective Service*
    t. *Sales & Related Occupations*
    u. *Transportation, Material Moving Occupations*
    v. *Something else not listed (please provide)*
12. What is your highest level of education?
    a. *Less than high school*
    b. *High school graduate*
    c. *Some college but no degree*
    d. *College graduate*
    e. *Graduate degree*
13. Would you be interested in participating in future research opportunities at Duke where you would share certain types of activity data from your smart device(s)?
    a. *Yes*
    b. *No*
    c. *Maybe*
14. Which types of activity data might you be comfortable sharing in future research activities?

	Yes	No	Maybe
Health tracking (blood oxygen, heart rhythm, women's health such as ovulation tracking, etc.)			
Fitness and workout monitoring (heart rate, step tracking, jogging, etc.)			
Sleep monitoring (sleep duration, sleep quality, etc.)			
Self-reported measures from health and fitness apps (mindfulness and mood, water intake, food logs, women's health, etc.)			

Supplementary Document 2: Demographic groups for each demographic factor

Gender:

1. 'Female',
2. 'Male',
3. 'Transgender Female',
4. 'Transgender Male',
5. 'Gender Fluid/Queer',
6. 'Non-binary',
7. 'Other Gender - Disclosed',
8. 'Other Gender - Not Disclosed'

Age/Generation:

1. 'Gen Z',
2. 'Millenials',
3. 'Generation X',
4. 'Baby Boomer',
5. 'Silent Generation',
6. 'Greatest Generation'

   *Generation categories were converted into age groups of 18-25, 26-41, 42-57, 58-76, 77-94, and 95+, respectively. Age group 77-94 and 95+ were combined into 77+ category, as we had a very limited representation in the 95+ category.

Race/Ethnicity:

1. 'Black or African American',
2. 'American Indian and/or Alaska Native',
3. 'Native Hawaiian and/or other Pacific Islander',
4. 'Asian or Asian American',
5. 'Hispanic',
6. 'White or Caucasian',
7. 'Another race / ethnicity',
8. 'Prefer not to answer'

Education:

1. 'Less than high school',
2. 'High school graduate',
3. 'Some college but no degree',
4. 'College graduate',
5. 'Graduate degree'

Employment Status:

1. 'Employed full-time',
2. 'Employed part-time',
3. 'Retired, not looking for work',
4. 'Disabled, not able to work',
5. 'Not employed, but looking for work',

6. 'Not employed, not looking for work'

Supplementary Table 1. Comparison of smart device ownership among respondents from our study with Pew Research studies with nationally representative sample population

	Smartphone Ownership		Wearable Ownership	
	Pew[1]	DHL	Pew[2]	DHL
**Total**	85%	98%	21%	59%
**Men**	85%	98%	18%	53%
**Women**	85%	98%	25%	62%
**White**	85%	98%	20%	60%
**Black**	83%	98%	23%	56%
**Hispanic**	85%	100%	26%	67%
**High school or less**	75%	99%	15%	39%
**Some college**	89%	97%	25%	56%
**College graduate**	93%	98%	27%	61%

Pew: Pew Research Studies

DHL: Duke Health Listens (Our Study)

References:

1 NW 1615 L. St, Washington S 800, Inquiries D 20036 U-419-4300 | M-857-8562 | F-419-4372 | M. Mobile Fact Sheet. Pew Res. Cent. Internet Sci. Tech. https://www.pewresearch.org/internet/fact-sheet/mobile/ (accessed Jan 5, 2023).

2 NW 1615 L. St, Suite 800Washington, Inquiries D 20036USA202-419-4300 | M-857-8562 | F-419-4372 | M. About one-in-five Americans use a smart watch or fitness tracker. Pew Res. Cent. https://www.pewresearch.org/fact-tank/2020/01/09/about-one-in-five-americans-use-a-smart-watch-or-fitness-tracker/ (accessed Sept 16, 2020).
