# Supplementary material for: Assessment of ownership of smart devices and the acceptability of digital health data sharing
Source: NPJ Digit Med. 2024 Feb 22;7:44. doi: 10.1038/s41746-024-01030-x (PMC10883993; doi:10.1038/s41746-024-01030-x)
Supplement: Supplementary file 2 — Reporting Summary [file 41746_2024_1030_MOESM2_ESM.pdf]

nature portfolio

Corresponding author(s): Jessilyn Dunn

Last updated by author(s): Jan 31, 2024

Reporting Summary

Nature Portfolio wishes to improve the reproducibility of the work that we publish. This form provides structure for consistency and transparency in reporting. For further information on Nature Portfolio policies, see our Editorial Policies and the Editorial Policy Checklist.

Statistics

For all statistical analyses, confirm that the following items are present in the figure legend, table legend, main text, or Methods section.

n/a	Confirmed	
☐	☒	The exact sample size (*n*) for each experimental group/condition, given as a discrete number and unit of measurement
☐	☒	A statement on whether measurements were taken from distinct samples or whether the same sample was measured repeatedly
☐	☒	The statistical test(s) used AND whether they are one- or two-sided<br>*Only common tests should be described solely by name; describe more complex techniques in the Methods section.*
☐	☒	A description of all covariates tested
☐	☒	A description of any assumptions or corrections, such as tests of normality and adjustment for multiple comparisons
☐	☒	A full description of the statistical parameters including central tendency (e.g. means) or other basic estimates (e.g. regression coefficient) AND variation (e.g. standard deviation) or associated estimates of uncertainty (e.g. confidence intervals)
☐	☒	For null hypothesis testing, the test statistic (e.g. *F*, *t*, *r*) with confidence intervals, effect sizes, degrees of freedom and *P* value noted<br>*Give P values as exact values whenever suitable.*
☒	☐	For Bayesian analysis, information on the choice of priors and Markov chain Monte Carlo settings
☒	☐	For hierarchical and complex designs, identification of the appropriate level for tests and full reporting of outcomes
☐	☒	Estimates of effect sizes (e.g. Cohen's *d*, Pearson's *r*), indicating how they were calculated

Our web collection on statistics for biologists contains articles on many of the points above.

Software and code

Policy information about availability of computer code

Data collection	We conducted a survey study among a representative groups of patients from the Duke University Health System (DUHS), called Duke Health Listens (DHL). DHL is an online community (with 3021 members at the time of our study, 95% of whom are patients from DUHS), where the members can offer direct feedback via online surveys on ways to improve the patient experience, ideas for new services or offerings, how to enhance DUHS’s online platforms, and feedback on health care messaging and marketing programs. We launched this survey study on January 18, 2022, and concluded it on January 30, 2022.
Data analysis	All analyses were conducted using Python version 3.7.4 through Jupyter notebooks (Jupyter notebook 6.0.1). The visualizations were generated using the Seaborn library in Python version 3.7.4.<br>The code used for this manuscript is available on the Big-Ideas-Lab (BIL) GitHub repository (https://github.com/Big-Ideas-Lab/bil-dhl-survey-analysis).

For manuscripts utilizing custom algorithms or software that are central to the research but not yet described in published literature, software must be made available to editors and reviewers. We strongly encourage code deposition in a community repository (e.g. GitHub). See the Nature Portfolio guidelines for submitting code & software for further information.

Data

Policy information about availability of data

All manuscripts must include a data availability statement. This statement should provide the following information, where applicable:

- Accession codes, unique identifiers, or web links for publicly available datasets
- A description of any restrictions on data availability
- For clinical datasets or third party data, please ensure that the statement adheres to our policy

The de-identified survey dataset generated and/or analyzed for the current study will be submitted 1 year from the publication date to the Big-Ideas-Lab (BIL) GitHub repository (https://github.com/Big-Ideas-Lab/bil-dhl-survey-analysis) under the title BigIdeasLab_DHL_Survey_Study_1.

Research involving human participants, their data, or biological material

Policy information about studies with human participants or human data. See also policy information about sex, gender (identity/presentation), and sexual orientation and race, ethnicity and racism.

Reporting on sex and gender	Nearly 64% participants included in the analysis were female. Gender was self reported by the participants.
Reporting on race, ethnicity, or other socially relevant groupings	Nearly 60% respondents identified as white and 6% identified as Hispanic. Race and ethnicity was self reported by the participants.
Population characteristics	1,368 members (of 3,021 members during the time of data collection) of the Duke Health Listen (DHL) patient advisory group responded to our surveys between January 18-30, 2022. A total of 871 (64%) were female, 826 (60%), identified as white, 390 (29%) identified as Black, 60 (4%) identified as Asian, 78 (6%), identified as Hispanic, and about half of the respondents (52%) were age 58 and above.
Recruitment	The survey was sent out to all DHL patient advisory group members (N=3021) between January 18-30, 2022 and we received responses from 1,368 individuals.
Ethics oversight	The participants of the Duke Health Listens (DHL) community provided their informed e-consent (using the Alida platform, Duke Health) when they joined this community to participate in studies organized by DHL. No separate consent was acquired for this specific purpose. The study was determined to be exempt from Institutional Review Board review by the Duke Health Institutional Review Board (Protocol ID: Pro00115157). The survey was prepared in collaboration with the DHL leadership. The DHL leadership conducted the survey study and provided the de-identified responses to the study team upon the completion of the survey.

Note that full information on the approval of the study protocol must also be provided in the manuscript.

Field-specific reporting

Please select the one below that is the best fit for your research. If you are not sure, read the appropriate sections before making your selection.

☐ Life sciences ☒ Behavioural & social sciences ☐ Ecological, evolutionary & environmental sciences

For a reference copy of the document with all sections, see nature.com/documents/nr-reporting-summary-flat.pdf

Behavioural & social sciences study design

All studies must disclose on these points even when the disclosure is negative.

Study description	Online survey to understand smart device ownership and usage, and participants' willingness to share data collected by smart devices for research purposes.
Research sample	The survey was collected from a large and diverse sample of patients from the Duke University Health System (DUHS), called Duke Health Listens (DHL). DHL is an online community (with 3021 members at the time of our study, 95% of whom are patients from DUHS), where the members can offer direct feedback via online surveys on ways to improve the patient experience, ideas for new services or offerings, how to enhance DUHS's online platforms, and feedback on health care messaging and marketing programs. Only adults aged 18 and older are allowed to be DHL advisors, and participation is not restricted to past or current DUHS patients
Sampling strategy	DHL is an online community (with 3021 members at the time of our study, 95% of whom are patients from DUHS) where the members can offer direct feedback via online surveys. The survey was sent out to all DHL patient advisory group members (N=3021) between January 18-30, 2022; 1,368 responded (45% response rate).
Data collection	Data was collected using online surveys distributed to DHL members. The DHL leadership conducted the survey study and provided the de-identified responses to the study team upon the completion of the survey.

Timing	The survey was launched on January 18, 2022 and was concluded on January 30, 2022.
Data exclusions	Data from all participants who responded were included in the analysis.
Non-participation	The survey was sent out to all DHL patient advisory group members (N=3021) and 1,368 members responded.
Randomization	No randomization was performed.

Reporting for specific materials, systems and methods

We require information from authors about some types of materials, experimental systems and methods used in many studies. Here, indicate whether each material, system or method listed is relevant to your study. If you are not sure if a list item applies to your research, read the appropriate section before selecting a response.

Materials & experimental systems

n/a	Involved in the study
☒	☐ Antibodies
☒	☐ Eukaryotic cell lines
☒	☐ Palaeontology and archaeology
☒	☐ Animals and other organisms
☒	☐ Clinical data
☒	☐ Dual use research of concern
☒	☐ Plants

Methods

n/a	Involved in the study
☒	☐ ChIP-seq
☒	☐ Flow cytometry
☒	☐ MRI-based neuroimaging

Plants

Seed stocks	N/A
Novel plant genotypes	N/A
Authentication	N/A
